# Effect of Metformin on Anthropometric Measurements and Hormonal and Biochemical Profile in Patients with Prediabetes

**DOI:** 10.1155/2021/8275303

**Published:** 2021-12-14

**Authors:** Mustafa Safiah, Dana Hyassat, Yousef Khader, Oraib Farahid, Anwar Batieha, Mohammed El-Khateeb, Kamel Ajlouni

**Affiliations:** ^1^Department of Endocrinology, The National Center for Diabetes, Endocrinology, and Genetics, The University of Jordan, Amman, Jordan; ^2^Department of Public Health, Jordan University of Science and Technology (JUST), Irbid, Jordan

## Abstract

**Objectives:**

Metformin is the most widely preferred first-line oral antidiabetic agent that results in clear benefits in blood sugar regulation and diabetes-related complications. This study is aimed at assessing the effect of metformin on anthropometric, hormonal, and biochemical parameters in patients with prediabetes or insulin resistance.

**Methods:**

A prepoststudy was conducted among 52 patients with prediabetes or insulin resistance who met the inclusion criteria. Weight, body mass index (BMI), and waist circumference were measured before and 12 months after metformin treatment. Serum concentrations of sex steroids, gonadotropins, and lipids were also assessed. Homeostasis model assessment (HOMA) index and quantitative sensitivity check (QUICKI) index scores were calculated before metformin treatment and after 12 months of use.

**Results:**

After 12 months of metformin treatment, female patients had significant reduction in weight, BMI, and waist circumference after adjusting for age. Metformin use for 12 months resulted in significant reduction in mean fasting blood glucose and HbA1c in females only. Total cholesterol decreased significantly among men only and serum HDL-C showed a significant rise among females only. Serum LDL-C and triglycerides did not change significantly in females and males. Our study did now significant changes in ACTH and cortisol levels in both females and males after metformin treatment. Metformin use resulted in significant increase in luteinizing hormone (LH) and progesterone levels in males, while it was associated with significant increase in prolactin, follicular stimulating hormone (FSH), and dehydroepiandrostenedione-sulphate (DHEA-S) levels and significant decrease in total testosterone level in females.

**Conclusion:**

Metformin treatment in females with prediabetes reduces BMI, waist circumference, fasting blood glucose, and HbA1c. The changes in the studied parameters differed significantly according to sex.

## 1. Introduction

Metformin is the most widely preferred first-line oral antidiabetic agent that results in clear benefits in blood sugar regulation and diabetes-related complications [1]. The mechanisms underlying these benefits include the improvement of insulin sensitivity in muscles and liver and the suppression of hepatic glucose production through the inhibition of glycogenolysis and gluconeogenesis. Beyond its glucose-lowering effect, metformin has been reported to decrease fatty liver disease and to reduce microvascular as well as macrovascular complications associated with type 2 diabetes [1, 2].

The use of metformin in women with gestational DM (GDM) resulted in less gestational weight gain and hypertension, fewer infants with macrosomia, and less neonatal hypoglycemia [3]. However, new follow-up information on offspring of mothers with GDM or polycystic ovary syndrome (PCOS) have provided conflicting results since metformin has the potential to inhibit the primary nutrient sensor in the placenta (mTOR) and mitochondrial activity resulting in relative nutrient restriction that might adversely affect fetal growth and potentially increase the risk of childhood obesity when the offspring is exposed to an obesogenic environment [3]. Insulin resistance is the beginning of many disorders such as PCOS, prediabetes, diabetes, and obesity in which metformin treatment is indicated. Therefore, insulin resistance is a disease that needs treatment to reduce the consequent complications related to diabetes and prediabetes [4].

In a systematic and meta-analysis review of 31 clinical trials, metformin treatment was found to increase ovulation, reduce serum androgen levels, and improve menstrual cycle in women with PCOS [5]. On the other hand, metformin theoretically with its antigrowth properties has been suggested as an adjuvant treatment for cancer [6]. In another meta-analysis of six studies, the treatment with metformin resulted in no significant change in body weight or other lipid profile criteria. This meta-analysis showed discrepant results about the influence of metformin on lipid profile as a whole. Some studies are in agreement with metformin reducing lipid profile and have reported a reduction, whereas the others have had contradictory values and results [7].

Type 2 diabetes is strongly associated with obesity, and this close relationship has led to the connotation “diabesity,” indicating the fact that the majority of individuals with type 2 diabetes are either obese or overweight. Obesity and dyslipidemia in people with type 2 diabetes are two major risk factors for coronary artery disease (CAD) and its related mortality [8]. Studies have reported that metformin is associated with reduced triglyceride (TG) and low-density lipoprotein cholesterol (LDL-C) and increased high-density lipoprotein cholesterol (HDL-C) levels [9]. It has also been reported that metformin increases nitric oxide production and can reduce the advanced glycation end products, intracellular reactive oxygen species production, and soluble vascular and intercellular cell-adhesion molecules in patients with type 2 diabetes independently of its glucose-lowering effect. As a result of all these functions, metformin could contribute to a lower risk of heart disease and mortality associated with its use [10, 11]. This study is aimed at assessing the effect of metformin on anthropometric, hormonal, and biochemical parameters in patients with prediabetes or insulin resistance.

## 2. Methods

### 2.1. Study Design

A prepostdesign was performed among patients with prediabetes or insulin resistance attending the National Center for Diabetes, Endocrinology, and Genetics (NCDEG). A total of 87 consecutive patients with prediabetes and/or insulin resistance not on metformin treatment and with normal renal and liver function patients were invited to participate in this study. The exclusion criteria included congestive heart failure, kidney or liver impairment, diabetes or its complications, and pregnant or lactating women. A total of 52 patients met the inclusion/exclusion criteria. Thirty eight of patients were females of whom 20 were postmenopausal, and 18 were premenopausal. Fourteen participants were males. The study variables were assessed and measured before administration of metformin and 12 months after the use of metformin (total dose was 1500 mg in 53.8% of patients and 2550 mg in 46.2% of patients). The study was approved by the ethics committee of NCDEG. All patients signed a consent form before entering the study.

### 2.2. Study Variables

Weight, height, and waist circumference were measured while the subjects were wearing light clothing and no shoes. Waist circumference was measured using a nonstretchable tape midway between the iliac crest and lower rib margin. Body mass index (BMI) was calculated by dividing weight (kg) by height in meters squared (m^2^).

Prediabetes was diagnosed using American Diabetes Association diagnostic criteria (2015) if the patient had fasting plasma glucose between 100 and 125 mg/dL or if the patient had impaired glucose tolerance (140-199 mg/dL) or if the patients had hemoglobin A1c (HbA1c) between 5.7% and 6.4% [12].

Fasting blood samples were drawn from a cannula inserted into the antecubital vein from each subject before the initiation of treatment with metformin and 12 months after treatment. For the menstruating women, the blood specimens were obtained in days 2 to 5 of the cycle. All biochemical measurements (luteinizing hormone [LH], follicle-stimulating hormone [FSH], thyroid-stimulating hormone [TSH], prolactin, estradiol, progesterone, total testosterone, free testosterone, sex hormone-binding globulin [SHBG], 17-OH-progesterone, dehydro-epiandrostenedione-sulphate [DHEA-S], androstenedione, cortisol, adrenocorticotropic hormone [ACTH], fasting insulin, FBS, high density lipoprotein cholesterol (HDL-C), low density lipoprotein cholesterol (LDL-C), total cholesterol, and triglycerides (TGs) were performed at the baseline and after 12 months of metformine use.

Fasting Homeostatic Modal Assessment (HOMA) index was calculated as (fasting insulin (mU/mL) × fasting glucose (mg/dL))/405). Quantitative Insulin Sensitivity Check (QUICKI) index was calculated as 1/log (fasting insulin (mU/mL) + fasting blood sugar (mg/dl)).

Serum concentration of glucose, total cholesterol, HDL-C, LDL-C, TGs, SHBG, DHEAS, and ACTH was assayed using cobas E-6000 (Roche Diagnostics, Manheim, Germany). HbA1c was measured using dedicated high pressure liquid chromatography (HPLC) method using D10 manufactured by BioRad (Bio-Rad Laboratories, Inc.USA). Serum insulin, FT4, TSH, LH, FSH, cortisol, prolactin, estradiol, and total testosterone were assayed using the ADVIA Centaur XPT Immunoassay System (Siemens Healthcare GmbH Erlangen Germany). Androstenedione, 17-OH-progesterone, and free testosterone were assayed using ELISA (Bio-Rad, Hercules, CA, USA). All measurements were performed according to the manufacturer instructions.

### 2.3. Statistical Analysis

The Statistical Package for Social Science (SPSS version 20) was used for data analysis. The chi-square test was used to analyze the differences between proportions. The general linear model repeated measures analysis was used to model changes in the studied parameters after adjusting for age and waist circumference. The changes in anthropometric measures for each sex were adjusted for age. The changes in other parameters were adjusted for age and baseline waist circumference. A *p* value of less than 0.05 was considered statistically significant.

## 3. Results

### 3.1. Participants' Characteristics

This study included 52 patients (38 females and 14 males) aged between 16 and 70 years with a mean (SD) age of 47.1 (11.7) year for females and 45.5 (7.7) year for males. The participants' characteristics are presented in Table 1. About 79% of males and 66% of females were obese, and 14.3% of males and 25% of females were overweight. About 55% of females and 57% of males had hypertension and 42% of females and 36% males had dyslipidemia.

### 3.2. Effect of Metformin Treatment on Anthropometric Measurements

The changes in anthropometric measurements differed significantly according to sex. The changes of these parameters after 12 months of metformin were not statistically significant in males after adjusting for age. However, the measurements changed significantly among females. After 12 months of metformin treatment, female patients had significant reduction in weight (from 82.5 to 77.6 kg, *p* value < 0.001 ), BMI (from 32.8 to 31.0 Kg/m^2^, *p* value = 0.001), and waist circumference (95.5 to 90.7 cm, *p* value = 0.001) after adjusting for age (Table 2).

### 3.3. Effect of Metformin Treatment on FBS, HbA1c, and Insulin Resistance

Metformin use for 12 months resulted in significant reduction in mean fasting blood glucose (from 101.7 to 84.1 mg/dl, *p* value = 0.017) and HbA1c (from 5.6% to 5.4%, *p* value = 0.001) in females only after adjusting for age and baseline waist circumference. Insulin resistance measured by homeostasis model assessment (HOMA) index and insulin sensitivity using the quantitative sensitivity check index (QUICKI) did not change significantly in both females and males (Table 2).

### 3.4. Effect of Metformin Treatment on Lipid Profile

Total cholesterol decreased significantly (from 196.1 to 186.3 mg/dl; *p* value = 0.028) among men only, and serum HDL-C showed a significant rise after 12 months of metformin use (from 29.8 to 46.8.0 mg/dl; *p* value < 0.001) among females only after adjusting for age and baseline waist circumference (Table 2). Serum LDL-C and triglycerides did not change significantly in females and males.

### 3.5. Effect of Metformin on Hormonal Profile

After 12 months of metformin treatment, there was a statistically significant increase in mean LH and progesterone levels in males (Table 3). Metformin treatment in premenopausal women resulted in a statistically significant increase in prolactin level (*p* value = 0.033) and FSH level (*p* value = 0.022) as well as DHEAS level (*p* value = 0.015) and a statistically significant decrease in total testosterone (*p* value = 0.032).

In postmenopausal women, we noticed a significant increase in prolactin level (*p* value = 0.043), LH levels (*p* value = 0.022), and FSH (*p* value = 0.008) as well as DHEAS (*p* value = 0.005) after metformin treatment (Table 4).

Our study did not show significant changes in ACTH and cortisol levels in both females and males after metformin treatment. A 12-month use of metformin resulted in a statistically significant increase in TSH level (from 2.6 to 3.2 *μ*IU/mL; *p* value = 0.008) among females only (Table 5).

## 4. Discussion

Our study showed that female patients who received metformin had a significant reduction in weight, BMI, and waist circumference. In agreement with our study, Valazquez et al. [13] reported a reduction in the mean Quetelet index (Kg/cm^2^ × 100) and waist hip ratio after metformin treatment. On the other hand, Kowalska et al. [14] found that 4 to 5 months of metformin treatment among obese patients with PCOS resulted in a significant reduction in BMI and percentage of body fat. One study showed that higher reduction in weight was achieved with higher doses of metformin [15]. On the other hand, other studies did not show changes in body weight after metformin therapy [16, 17].

Total cholesterol decreased significantly among men only, and serum HDL-C showed a significant rise after 12 months of metformin use among women. Moghetti et al. [18] found that metformin significantly increased HDL-C level in women with PCOS after 6 months. Additionally, Singh et al. [19] found that administration of metformin to 22 PCOS cases in a dose of 500 mg three times daily for 3 months was associated with a significant rise in HDL-C (*p* < 0.001), with all other lipid parameters showing no marked difference after metformin treatment. Moreover, Defronzo et al. [20] found that metformin treatment for 29 weeks significantly reduced plasma total cholesterol, LDL-cholesterol, and TG concentration.

We found that women treated with metformin showed a significant reduction in total testosterone (but not free testosterone) and a significant increase in DHEA-S, but there was no evidence of an increase in sex hormone binding globulin. The reduction in total testosterone might be partly explained by the decrease in insulin levels or by the direct effect of metformin on ovarian stroma.

Consistent with our finding, Thomas et al. [5] in a systematic review found that androgen DHEA-S was significantly increased in the metformin-treated group. Additionally, in a study conducted by Patel et al. [21] on postmenopausal women with previous PCOS and or insulin resistance, metformin reduced testosterone and free testosterone, but it did not modify DHEA-S. On the other hand, Jakubowicz et al. [22, 23] studies had reported a reduction in DHEA-S with metformin in contrast to the other trials. Moreover, Campagnoli et al. [24] concluded that the use of metformin resulted in a significant reduction in free testosterone with an insignificant increase in DHEA-S levels.

In another systemic review and meta-analysis, Barba et al. [25] found that metformin decreased the circulating levels of total testosterone, DHEAS, and androstenedione, along with increasing the circulating level of SHBG, but the results were not statistically significant for free testosterone.

Our study showed a significant rise in TSH level in euthyroid female patients treated with metformin for 12 months. Consistent with our finding, Diez et al. [26] also found that patients taking metformin exhibited significantly higher TSH levels than those without metformin treatment, but this significant relationship between TSH and metformin was lost when introducing some confounding variables into the model, such as goiter, BMI, macroangiopathy, and hyperlipidemia. Lupoli et al. [27] found that metformin induces a reduction in TSH levels in patients with overt and subclinical hypothyroidism, with no change in TSH levels in euthyroid patients. Fournier et al. [28] also showed that metformin's effect on lowering TSH was observed only in the group of patients with treated hypothyroidism, while there is no effect on TSH levels in euthyroid patients, as prescribed by Vigersky et al. [29].

In our study, metformin was shown to increase prolactin levels in females with a normal baseline level of prolactin. On the other hand, Krysiak et al. [30] found that metformin decreases prolactin levels only if given at high doses to patients with elevated prolactin levels. Finally, the use of metformin in our study did not demonstrate a change in ACTH level and cortisol level. However, metformin had no effects on 17 OH-progesterone, androstenedione, estradiol, SHBG, and T4 levels in both males and females enrolled in this study. The clinical significance of these findings needs further investigation.

One of the limitations of this study is the small sample size. A larger sample size is needed to assess the effects of metformin on the studied parameters.

## 5. Conclusions

Metformin treatment in females with prediabetes reduces BMI, waist circumference, fasting blood glucose and HbA1c. The changes in the studied parameters differed significantly according to sex.

## Figures and Tables

**Table 1:** Baseline characteristics of study participants.

Variable	Female (*n* = 38), *n* (%)	Male (*n* = 14), *n* (%)
Age (years) (mean ± SD)	47.1 ± 11.7	45.5 ± 7.7
Marital status		
Single	5 (13.2)	1 (7.1)
Married	33 (86.8)	13 (92.9)
Body mass index(BMI) (kg/m^2^)		
Normal	3 (9.4)	1 (7.1)
Overweight	8 (25.0)	2 (14.3)
Obese	21 (65.6)	11 (78.6)
Physical activity		
Active	4 (10.5)	3 (21.4)
Not active	34 (89.5)	11 (78.6)
Smoking		
Current	4 (10.3)	9 (64.3)
Nonsmoker	35 (89.7)	5 (35.7)
Metformin dose		
1500 mg	21 (55.3)	7 (50.0)
2550 mg	17 (44.7)	7 (50.0)
Hypertension	21 (55.3)	8 (57.1)
Dyslipidemia	27 (71.1)	10 (71.4)
On statin	16 (42.1)	5 (35.7)

**Table 2:** The effect of metformin on metabolic profile in prediabetic participants.

Variable	Baseline	After 12 months	Adjusted *p* value (GLM)^∗^
	Mean ± SD	Mean ± SD	
Weight (kg)			
Female	82.5 ± 14.0	77.6 ± 13.4	<0.001
Male	96.3 ± 14.5	93.1 ± 15.6	0.279
Body mass index (BMI)			
Female	32.8 ± 6.5	31.0 ± 5.9	0.001
Male	32.5 ± 5.1	31.6 ± 5.6	0.253
Waist circumference (cm)			
Female	95.5 ± 10.2	90.7 ± 10.1	0.001
Male	105.6 ± 10.6	103.2 ± 12.8	0.290
Fasting blood sugar (mg/dl)			
Female	101.7 ± 13.8	84.1 ± 32.8	0.017
Male	112.3 ± 15.7	106.0 ± 9.0	0.300
Hemoglobin A1c			
Female	5.6 ± 0.4	5.4 ± 0.4	0.001
Male	5.7 ± 0.4	5.6 ± 0.3	0.041
Insulin			
Female	13.3 ± 5.6	11.8 ± 5.4	0.038
Male	17.1 ± 8.9	13.6 ± 7.8	0.366
Quantitative Sensitivity Check (QUICKI)			
Female	0.3 ± 0.02	0.4 ± 0.11	0.062
Male	0.3 ± 0.03	0.3 ± 0.03	0.730
Homeostasis model assessment (HOMA)			
Female	3.5 ± 1.9	3.0 ± 1.4	0.092
Male	4.7 ± 2.4	3.7 ± 2.2	0.606
Total cholesterol			
Female	192.7 ± 44.2	183.9 ± 42.4	0.182
Male	196.1 ± 19.2	186.3 ± 46.1	0.028
Low density lipoprotein cholesterol (mg/dl)			
Female	119.4 ± 37.0	110.5 ± 30.2	0.157
Male	117.0 ± 32.2	104.2 ± 33.8	0.306
High-density lipoprotein cholesterol (mg/dl)			
Female	29.8 ± 9.1	46.8 ± 11.9	<0.001
Male	27.9 ± 13.0	40.2 ± 12.0	0.077
Triglyceride (mg/dl)			
Female	141.2 ± 60.6	133.4 ± 62.2	0.124
Male	158.9 ± 54.5	152.1 ± 53.2	0.569

^∗^The changes in anthropometric variables are adjusted for age, and the changes in other variables are adjusted for age and waist circumference at the baseline. GLM: general linear model repeated measures analysis.

**Table 3:** Effect of metformin on pituitary and sex hormones in prediabetic males (*N* = 14).

	Baseline, mean ± SD	After 12 months, mean ± SD	*p* value
Prolactin	7.3 ± 2.4	7.9 ± 2.2	0.105
Luteinizing hormone (LH)	3.9 ± 1.7	5.1 ± 1.85	0.003
Follicle-stimulating hormone (FSH)	5.8 ± 5.3	6.2 ± 4.3	0.368
Free testosterone	10.6 ± 2.3	11.5 ± 5.0	0.481
Total testosterone	3.5 ± 1.0	3.7 ± 1.4	0.597
Estradiol	28.0 ± 6.6	34.0 ± 12.3	0.144
Progesterone	0.26 ± 0.22	0.32 ± 0.22	0.028
Sex hormone-binding globulin (SHBG)	31.6 ± 18.3	31.1 ± 14.1	0.852
17-OH progesterone	2.9 ± 1.4	3.2 ± 1.4	0.068
Androstendione	2.2 ± 0.9	2.3 ± 1.1	0.598
Dehydroepiandrostenedione-sulphate (DHEAS)	186.1 ± 94.4	198.2 ± 103.5	0.489

**Table 4:** Effect of metformin on pituitary and sex hormones in menopausal and nonmenopausal prediabetic (*N* = 38).

	Not menopausal			Menopausal		
	Baseline, mean ± SD	After 12 months, mean ± SD	*p* value	Baseline, mean ± SD	After 12 months, mean ± SD	*p* value
Prolactin	8.9 ± 4.9	10.9 ± 6.2	0.033	6.1 ± 2.3	7.1 ± 3.0	0.043
Luteinizing hormone (LH)	4.6 ± 2.4	5.2 ± 3.1	0.455	28.5 ± 18.0	34.8 ± 22.7	0.022
Follicle-stimulating hormone (FSH)	6.2 ± 3.3	8.1 ± 4.7	0.022	58.8 ± 35.2	65.3 ± 33.0	0.008
Free testosterone	1.3 ± 0.6	1.2 ± 0.5	0.271	1.2 ± 0.4	1.3 ± 0.6	0.537
Total testosterone	0.5 ± 0.2	0.42 ± 0.1	0.032	0.5 ± 0.4	0.47 ± 0.6	0.101
Estradiol	42.9 ± 25.1	37.0 ± 17.1	0.491	23.6 ± 19.6	21.3 ± 15.0	0.505
Progesterone	0.41 ± 0.2	0.38 ± 0.2	0.548	0.39 ± 0.9	0.17 ± 0.14	0.303
Sex hormone-binding globulin (SHBG)	46.3 ± 22.4	45.7 ± 21	0.892	53.7 ± 44.2	57.7 ± 44.4	0.214
17-OH progesterone	1.6 ± 0.8	1.8 ± 1.4	0.484	1.3 ± 0.9	1.7 ± 1.5	0.201
Androstendione	1.8 ± 0.7	1.8 ± 0.6	0.859	1.67 ± 0.7	1.78 ± 0.8	0.529
Dehydroepiandrostenedione-sulphate (DHEAS)	120.7 ± 65.4	144.6 ± 85.8	0.015	89.7 ± 53.8	106.8 ± 58.3	0.005

**Table 5:** Impact of effect on other hormones in prediabetic participants (*N* = 52).

Variable	Baseline	After 12 months	Adjusted *p* value (GLM)^∗^
	Mean ± SD	Mean ± SD	
Adrenocorticotropic hormone (ACTH)			
Female	9.6 ± 7.5	14.0 ± 10.4	0.054
Male	11.3 ± 10.7	12.5 ± 6.7	0.883
Cortisol			
Female	14.8 ± 6.0	14.7 ± 5.4	0.869
Male	15.6 ± 4.7	15.0 ± 4.9	0.542
Thyroid stimulating hormone (TSH)			
Female	2.6 ± 1.2	3.2 ± 1.5	0.008
Male	2.6 ± 1.4	2.7 ± 1.3	0.733
Free thyroxine (FT4)			
Female	16.1 ± 2.0	16.3 ± 2.5	0.311
Male	15.6 ± 2.1	15.6 ± 2.4	0.876

^∗^The changes in the parameters are adjusted for age and waist circumference at the baseline. GLM: general linear model procedure.

## Data Availability

The SPSS data used to support the findings of this study are available from the corresponding author upon request.
